# Complete Genome Sequence of Escherichia coli Strain BL21

**DOI:** 10.1128/genomeA.00134-15

**Published:** 2015-03-19

**Authors:** Haeyoung Jeong, Hyun Ju Kim, Sang Jun Lee

**Affiliations:** a Super-Bacteria Research Center, Korea Research Institute of Bioscience and Biotechnology (KRIBB), Daejeon, Republic of Korea; b Biosystems and Bioengineering Program, University of Science and Technology (UST), Daejeon, Republic of Korea; c Infection and Immunity Research Center, KRIBB, Daejeon, Republic of Korea

## Abstract

Escherichia coli strain BL21 is one of the widely used bacterial hosts for high-level recombinant protein production and for other applications. Here, we present the complete genome sequence of a commercial version of the Escherichia coli BL21 strain.

## GENOME ANNOUNCEMENT

Escherichia coli BL21 and BL21(DE3), created by F. William Studier and Barbara A. Moffatt (1), are common laboratory strains for recombinant protein production. The lack of *lon* and *ompT* proteases, often regarded as common characteristics among B lineage, has driven the development of those strains for protein expression hosts. E. coli BL21(DE3), a derivative of BL21, is probably the most widely used in high-level expression of recombinant proteins, and it harbors a prophage DE3 derived from a bacteriophage λ, which carries the T7 RNA polymerase gene under the control of the *lac*UV5 promoter. E. coli BL21 has been routinely used for non-T7 expression, and it was also recently modified to produce a plasmid DNA vaccine, due to its better performance in high-cell-density fed-batch cultures compared to K-12 strains (2).

The genome sequence of E. coli BL21(DE3) was previously determined by our institute (3). In principle, the genome sequence of BL21 would be identical with that of BL21(DE3), except for the DE3 prophage. However, stock-to-stock variations may occur that can have serious implications for experiments (4). With E. coli BL21(DE3) genome information as the reference, the complete genome sequence of BL21 was constructed using only next-generation sequencing, and base-by-base genomic differences were identified.

E. coli BL21 strain was purchased from TaKaRa Bio (code number 9126, lot number K142). Genome sequencing was carried out at the Human Derived Material Center, Korea Research Institute of Bioscience and Biotechnology (KRIBB), using the Illumina HiSeq 2000 system. The complete genome sequence of BL21 was produced both by elaborated reference mapping and *de novo* assembly of 101-nt Illumina reads (47,123,524 paired-end reads from an ~150-bp library) using the CLC Genomics Workbench version 6.5.1 (CLC bio).

We had expected that a modified BL21(DE3) genome sequence (CP001509.3)—from which the prophage region was deleted, leaving one copy of the lambda attachment site (*attB*)—would serve as the best reference sequence. However, coverage analysis from the first mapping showed that the *attB* of BL21 was not empty but occupied by a 12.1-kb-long λ*B prophage as in E. coli REL606 (3), which was reported to be a common characteristic among the B lineage (5). A second trial of reference mapping, using a modified sequence having a λ*B prophage sequence, revealed another low-coverage region between the *ybhC* gene and the left boundary of the lambda attachment site*, attL*. By sequence comparison, the best matching sequence was identified from *de novo* assembled contigs and was 23 bp longer than the corresponding region from BL21(DE3). An updated reference sequence was then prepared by replacing the low coverage region and its neighbor (~200 bp), and mapping was carried out again. Uniform read coverage on the entire final reference sequence was confirmed by visual investigation, and no other difference was found by quality-based SNV/structural variation detection or by breakpoint analysis. Assembly using unmapped reads did not result in any contigs that can support the presence of BL21-specific regions. Genome annotation was carried out by the NCBI Prokaryotic Genome Automatic Annotation Pipeline (PGAAP) service.

### Nucleotide sequence accession number.

This whole-genome shotgun project has been deposited at DDBJ/EMBL/GenBank under the accession number CP010816.
